# Sex differences in the prevalence of metabolic dysfunction-associated steatotic liver disease in hospitalized patients: a single-center cross-sectional study

**DOI:** 10.3389/fendo.2026.1942008

**Published:** 2026-10-22

**Authors:** Zhemin Liu, Suosu Wei

**Affiliations:** 1 Department of Project Management and Technology Transfer, Guangxi Academy of Medical Sciences, the People’s Hospital of Guangxi Zhuang Autonomous Region, Nanning, Guangxi, China; 2 Clinical Research Center of Guangxi Academy of Medical Sciences, the People’s Hospital of Guangxi Zhuang Autonomous Region, Nanning, Guangxi, China

**Keywords:** cross-sectional study, MASLD, metabolic dysfunction, prevalence, sex

## Abstract

**Background:**

The influence of sex and age on the prevalence of metabolic dysfunction-associated steatotic liver disease (MASLD) among hospitalized patients remains unclear.

**Methods:**

This cross-sectional study included 136,816 hospitalized adults (2014–2023) at the People’s Hospital of Guangxi Zhuang Autonomous Region. Hepatic steatosis was confirmed by ultrasonography. Sex-specific prevalence was analyzed across younger (18–50 years) and older (51–80 years) age groups.

**Results:**

MASLD prevalence was 28.8% (39,431/136,816). MASLD patients were older than those without MASLD (median 58 vs. 53 years; *P* < 0.001). Within the hospitalized population, age-stratified analysis revealed sex-specific differences in the odds of MASLD reversal. Women aged 18–50 had a lower prevalence of MASLD than men (OR< 1, P < 0.001), whereas women aged 51-80 had a higher prevalence (OR> 1, P < 0.001). After adjustment for age, calendar year, and sex × age interaction, the sex difference remained significant (*P* < 0.001). Women aged 18–50 years had lower adjusted odds of MASLD than men (aOR = 0.50, 95% CI: 0.48–0.52), whereas women aged 51–80 years had higher adjusted odds (aOR = 1.69, 95% CI: 1.64–1.74). Comparing older women (aged 51–80) with younger women (aged 18–50), the prevalence of MASLD was significantly higher among older women (*P* < 0.001). Among all patients, older hospitalized patients also exhibited more pronounced metabolic abnormalities, including elevated BMI (81.8% vs. 44.1%), elevated blood pressure (72.2% vs. 61.0%), and elevated triglycerides (44.6% vs. 20.8%) (all *P* < 0.001).

**Conclusion:**

Early MASLD screening should be prioritized in hospitalized women aged 50 years and older and hospitalized men under 50 years, particularly those with metabolic prevalence factors, to enable timely intervention and reduce the development and progression of MASLD.

## 1 Introduction

Metabolic dysfunction-associated steatotic liver disease (MASLD) (formerly known as nonalcoholic fatty liver disease) represents the most prevalent chronic liver disease globally, encompassing a spectrum of liver conditions ranging from simple steatosis to hepatocellular carcinoma (1). It is closely associated with insulin resistance and metabolic syndrome (2). Currently, MASLD affects over one-third of the global adult population, with particularly high prevalence among individuals with metabolic disorders such as type 2 diabetes and obesity (3). A predictive modeling study indicates an exponential growth in its disease burden, projecting a prevalence rate of 33.5% among adults (≥15 years) by 2030 (4). Consequently, MASLD has become a major public health concern, imposing substantial clinical and economic burdens on healthcare systems worldwide (5). Furthermore, MASLD patients often experience impaired health-related quality of life (HRQL) and may face stigmatization (6). Given this burden, effective prevention and treatment strategies for MASLD are urgently needed.

MASLD exhibits sexual dimorphism, with age-related hormonal changes representing a potential turning point in disease progression. Overall prevalence is higher in males than in females (7). However, this trend reverses after women reach approximately 50 years of age. The transition to menopause involves not only the loss of ovarian estrogen production but also the progressive physiological changes associated with aging, which together may disrupt organismal homeostasis and predispose women to a cluster of metabolic disorders (8). Multiple studies consistently report that MASLD prevalence increases markedly in women aged ≥50 years, approaching or surpassing prevalence in age-matched men (9). Analyses of the National Health and Nutrition Examination Survey indicate that although the overall prevalence is higher in men, the rate of increase is substantially faster in women (10). Even after adjustment for age and body mass index (BMI), the elevated prevalence in women aged ≥50 years remains statistically significant (11–13). Female patients with MASLD also exhibit poorer clinical outcomes, with MASLD-related mortality appearing to increase more rapidly in women than in men (14).

Beyond insulin resistance, multiple endocrine axes—including growth hormone, thyroid, adrenal, and sex hormone pathways—have been implicated in the pathogenesis of MASLD (15). Estrogen plays a central role in the regulation of hepatic metabolism (16). Numerous studies suggest that age-related hormonal changes may promote the onset and progression of MASLD (16–18). Although obesity prevalence is higher among women in some populations, women aged <50 years appear partially protected against MASLD (19). This protection likely reflects sex-specific regulation of metabolic and inflammatory pathways; whereby hepatic metabolism is modulated by sex hormones—particularly estrogen—and influenced by reproductive demands (20–23). Liver diseases display sex-specific prevalence patterns (2, 24) and are linked to female reproductive dysfunction (20, 25). Clinical data indicate that reduced ovarian estrogen secretion during menopause may contribute to multisystem metabolic dysfunction, including hepatic lipid accumulation, insulin resistance, and fibrosis, as well as extrahepatic metabolic alterations in adipose tissue and skeletal muscle (16). However, because menopausal status was not directly measured in this study, we did not assess menopause as a variable; instead, we used age ≥50 years as a proxy.

With improvements in living standards and more widespread use of ultrasonography, the number of diagnosed fatty liver cases in China has increased steadily in recent years (26). However, domestic studies on sex-specific MASLD prevalence have largely focused on the general population, and direct evidence on the interaction between sex, age, and metabolic factors within specific age strata remains limited. Hospitalized patients, who often present with more pronounced metabolic abnormalities and a higher burden of comorbidities, may provide valuable insights into the sex- and age- specific patterns of MASLD in a high-risk clinical setting. Therefore, we conducted a cross-sectional study to analyze the relationship between sex, age, and MASLD prevalence in a hospitalized population at a tertiary medical center.

## 2 Materials and methods

### 2.1 Study population and study design

This cross-sectional study included adult patients (18–80 years) who underwent liver ultrasound examinations during hospitalization at the People’s Hospital of Guangxi Zhuang Autonomous Region from January 1, 2014, to September 7, 2023 (Trial registration: https://www.chictr.org.cn/; ChiCTR2400088388). Data were obtained from the hospital’s active health-management platform, which integrates comprehensive clinical information (outpatient visits, hospitalization records, and physical examination data) and related diagnostic and therapeutic records. The system aggregates treatment records, laboratory and imaging reports, and electronic medical records from outpatient, inpatient, and physical examination services, enabling centralized access and automatic data integration during patient encounters.

Initially, 255,187 hospitalized patients who underwent liver ultrasonography were identified. For patients with repeated hospitalizations or ultrasound examinations, only the first ultrasound examination at admission was included, and subsequent examinations were not analyzed. The date of the first ultrasound examination at admission was defined as the index date. Thus, the analytic dataset included one observation per unique patient. Patients missing data required to define metabolic dysfunction were excluded, including missing fasting glucose (n = 64,062), systolic blood pressure (SBP) (n = 18,606), diastolic blood pressure (DBP) (n = 2,192), triglycerides (TG) (n = 32,950), and high-density lipoprotein cholesterol (HDL-C) (n = 3). Overall, 118,371 of 255,187 patients (46.4%) were excluded due to missing data required for MASLD classification. Among the included participants, missing rates for the remaining variables were low: ALT/AST/GGT (1.3% each), platelet count (PLT) (13%), and albumin (1.3%). To assess potential selection bias, we compared the included and excluded groups using standardized differences (SMDs) and examined the distribution of excluded participants across calendar years, sex, and age groups (**Supplementary Tables S1**–**S3**). These analyses demonstrated that the excluded group was broadly comparable to the included group, with the vast majority of SMDs below 0.1. **Figure 1** illustrates the detailed inclusion and exclusion process. Ultimately, 136,816 patients were included in the analysis: 39,431 in the MASLD group and 97,385 in the non-MASLD group. The study was conducted in accordance with the guidelines of the Declaration of Helsinki (6th revision, 2008) and was approved by the Ethics Committee of the People’s Hospital of Guangxi Zhuang Autonomous Region (Approval No.: KY-IIT-2024-02). The requirement for individual informed consent was waived due to the use of anonymized data.

**Figure 1:**
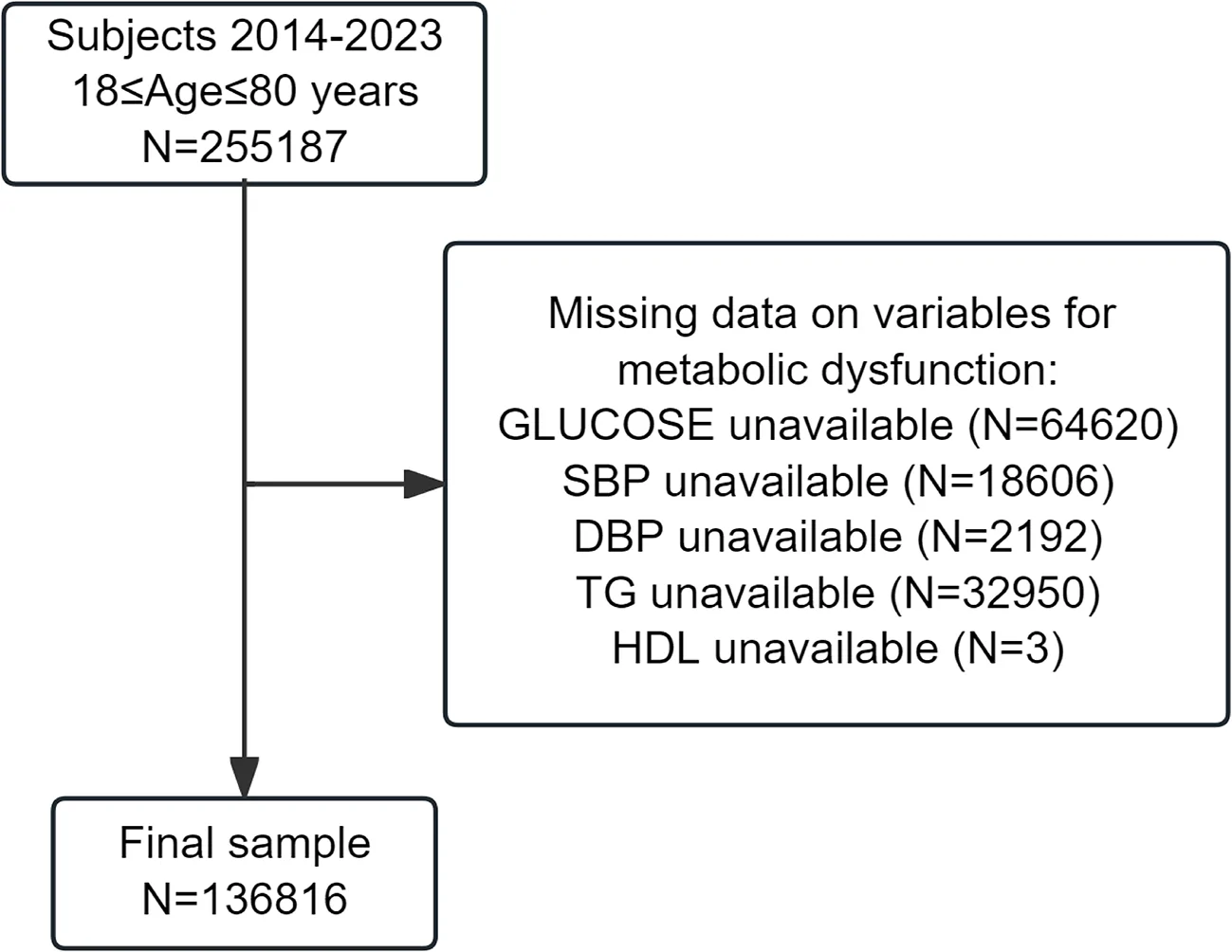
Flow chart of the study participants. SBP, systolic blood pressure; DBP, diastolic blood pressure; TG, triglycerides; HDL, high-density lipoprotein; BMI, body mass index.

### 2.2 Ascertainment of MASLD

All participants with ultrasound evidence of hepatic steatosis were evaluated according to the recent Delphi consensus criteria (27). Due to data unavailability in our electronic medical records, the following parameters were not included: waist circumference, HbA1c, use of antihypertensive medication, and use of lipid-lowering therapy. According to the 2023 Delphi consensus, alcohol consumption is not an exclusion criterion for MASLD. We therefore assessed alcohol intake only as current drinking status (yes/no) and did not exclude participants from MASLD classification based on alcohol use. However, detailed quantitative alcohol data were unavailable, so we could not distinguish MetALD from MASLD. Patients with HBV or HCV infection were excluded from the MASLD group. These items were therefore omitted from the diagnostic criteria below. The absence of these parameters may lead to underdiagnosis or misclassification, which is acknowledged as a limitation. In the absence of other liver diseases or viral hepatitis, participants were diagnosed with MASLD if they met at least one of the following criteria:

- BMI ≥23 kg/m^2^ (Asian criteria);
- Fasting plasma glucose ≥5.6 mmol/L, or a previous diagnosis of type 2 diabetes;
- Blood pressure ≥130/85 mmHg;
- Triglycerides (TG) ≥1.7 mmol/L;
- HDL-C <1.0 mmol/L (males) or <1.3 mmol/L (females).

### 2.3 Variables

Baseline characteristics were comprehensively assessed. Participants were stratified by MASLD status and further subdivided by sex (71,690 females and 65,126 males) and by age (18–50 years and 51–80 years). BMI was calculated as weight in kilograms divided by height in meters squared (kg/m²). SBP and DBP were measured clinically. Laboratory tests included aspartate aminotransferase (AST), alanine aminotransferase (ALT), gamma-glutamyl transferase (GGT), triglycerides (TG), HDL-C, fasting plasma glucose, serum albumin, and platelet count (PLT). Following the MASLD criteria (27), binary variables were created for elevated blood pressure, elevated BMI, elevated triglycerides, low HDL-C, and elevated fasting glucose. The study evaluated the prevalence of comorbidities, including type 2 diabetes (T2DM), chronic kidney disease (CKD), cardiovascular disease (CVD). Lifestyle factors included smoking and alcohol consumption. Alcohol use was recorded as current drinking status (yes/no) and was not used as an exclusion criterion for MASLD. The Fibrosis-4 index (FIB-4) was used to assess liver fibrosis, AST (IU/L) ALT (IU/L) (28). Participants were classified into three risk groups: FIB-4 < 1.3 (low risk), FIB-4 1.3–2.67 (intermediate risk), and FIB-4 > 2.67 (high risk).

### 2.4 Statistical analysis

Categorical variables were presented as frequencies and percentages, and continuous variables as medians with interquartile ranges (IQRs). Baseline characteristics of the MASLD and non-MASLD groups were compared using the Mann-Whitney U test for continuous variables and the chi-square test for categorical variables. Stratified comparative analyses of demographic characteristics, metabolic indicators, and comorbidities were performed across different age groups (18–50 years and 51–80 years) and by sex (male and female). For cross-sectional comparisons, sex differences in prevalence within the same calendar year or age stratum were assessed using the chi-square test.

The association between sex and MASLD prevalence was quantified using unadjusted binary logistic regression, reporting odds ratios (ORs) with 95% confidence intervals (CIs). An OR > 1 indicates higher odds in females compared with males (reference), whereas an OR < 1 indicates lower odds. To account for potential confounding and to formally test whether the sex difference varied by age, a multivariable logistic regression model was constructed with MASLD as the dependent variable and sex, age (centered at 50 years), calendar year, and a sex × age interaction term as independent variables. Adjusted odds ratios (aORs) with 95% CIs were reported. The sex × age interaction was tested using the Wald test. When the interaction was significant, adjusted ORs for sex were also calculated separately for the 18–50 and 51–80 year age groups.

To further explore whether the sex difference was independent of metabolic risk factors, additional multivariable logistic regression models stratified by age group were performed, adjusting for age, BMI, glucose, triglycerides, HDL-C, T2DM, CVD, and calendar year. The results are presented in **Supplementary Table S8**.

Temporal trends were evaluated using simple linear regression with the calendar year as the independent variable and MASLD prevalence as the dependent variable to obtain *P* for trend. To quantify annual changes in sex-specific prevalence differences, generalized linear models (binary logistic regression) were fitted separately for the overall population, the 18–50-year age group, and the 51–80-year age group; a year × sex interaction term was included to test whether sex differences in MASLD prevalence changed over time. In addition, multivariable logistic regression models stratified by age group were performed, adjusting for age, BMI, glucose, triglycerides, HDL-C, T2DM, CVD, and calendar year.

To further characterize the age-related trend in male MASLD prevalence, we used Joinpoint time-series analysis (Joinpoint Regression Program, version 5.4.1, National Cancer Institute) to identify points where the trend changed significantly (joinpoints) and to estimate the annual percent change (APC) for each age segment. Age was used as the independent variable. The number of joinpoints was selected using the weighted Bayesian Information Criterion (WBIC), and the average annual percent change (AAPC) was also calculated.

To assess potential selection bias due to missing data, we compared the baseline characteristics of the included and excluded groups using standardized differences (SMDs) and examined the distribution of excluded participants across calendar years, sex, and age groups (**Supplementary Tables S1**–**S3**). Given the substantial proportion of excluded participants due to missing metabolic data, sensitivity analyses were conducted to assess potential selection bias (**Supplementary Tables S1**–**S3**). All reported *P* values are two-tailed, with *P* < 0.05 considered statistically significant. Statistical analyses were conducted using SPSS version 25, and data visualization was performed using R version 4.6.1 with the ggplot2 package.

## 3 Results

### 3.1 Baseline characteristics

After applying the exclusion criteria, 136,816 participants were included in the analysis, of whom 39,431 (28.8%) were diagnosed with MASLD. Participants with MASLD were more likely to be older, female, and to have T2DM or CVD (all *P* < 0.001). Compared with participants without MASLD, those with MASLD had higher BMI, blood pressure, fasting plasma glucose, ALT, AST, GGT, and platelet count. They also had higher TG levels, lower HDL-C levels, and lower prevalence of CKD and smoking (all *P* < 0.001; **Table 1**).

**Table 1:** Baseline characteristics of the study subjects.

Variables	Total(n=136816)		*P* value	Female (n = 71690)		*P* value	Male (n=65126)		*P* value
	Non-MASLD (n=97385)	MASLD (n=39431)		Non-MASLD (n=50673)	MASLD (n=21017)		Non-MASLD (n=46712)	MASLD (n=18414)	
Age (years)	53.0 (40.0-64.0)	58.0 (48.0-66.0)	**<0.001**	49.0 (36.0-61.0)	59.0 (50.0-66.0)	**<0.001**	57.0 (46.0-66.0)	57.0 (46.0-65.0)	**<0.001**
Female (%)	50673 (52.0)	21017 (53.3)	**<0.001**	N/A	N/A	N/A	N/A	N/A	N/A
SBP (mmHg)	126.0 (113.0-142.0)	135.0 (123.0-150.0)	**<0.001**	121.0 (110.0-137.0)	135.0 (121.0-149.0)	**<0.001**	131.0 (119.0-147.0)	136.0 (124.0-150.0)	**<0.001**
DBP (mmHg)	76.0 (69.0-85.0)	81.0 (74.0-89.0)	**<0.001**	75.0 (68.0-82.0)	80.0 (72.0-87.0)	**<0.001**	79.0 (71.0-88.0)	83.0 (75.0-91.0)	**<0.001**
BMI (kg/m ^2^)	22.4 (20.3-24.6)	25.6 (23.7-27.7)	**<0.001**	21.9 (20.0-24.1)	25.3 (23.4-27.6)	**<0.001**	22.9 (20.7-25.1)	25.9 (24.1-28.0)	**<0.001**
AST (IU/L)	20.0 (17.0-26.0)	21.0 (18.0-26.0)	**<0.001**	19.0 (16.0-24.0)	20.0 (17.0-25.0)	**<0.001**	22.0 (18.0-30.0)	22.0 (18.0-27.0)	**<0.001**
ALT (IU/L)	15.0 (11.0-24.0)	19.0 (14.0-28.0)	**<0.001**	13.0 (10.0-18.0)	17.0 (13.0-24.0)	**<0.001**	19.0 (13.0-30.0)	23.0 (16.0-34.0)	**<0.001**
GGT (IU/L)	21.0 (15.0-38.0)	29.0 (20.0-45.0)	**<0.001**	16.0 (12.0-23.0)	24.0 (18.0-34.0)	**<0.001**	31.0 (21.0-56.0)	37.0 (26.0-57.0)	**<0.001**
Triglycerides (mmol/L)	1.1 (0.8-1.6)	1.6 (1.2-2.3)	**<0.001**	1.1 (0.8-1.5)	1.6 (1.2-2.2)	**<0.001**	1.2 (0.9-1.7)	1.7 (1.2-2.4)	**<0.001**
HDL-C (mmol/L)x	1.2 (1.0-1.5)	1.1 (0.9-1.3)	**<0.001**	1.3 (1.1-1.6)	1.2 (1.0-1.4)	**<0.001**	1.1 (0.9-1.3)	1.0 (0.9-1.2)	**<0.001**
Glucose (mmol/L)	4.6 (4.2-5.0)	5.0 (4.5-5.8)	**<0.001**	4.5 (4.2-5.0)	5.0 (4.5-5.7)	**<0.001**	4.7 (4.2-5.2)	5.0 (4.5-5.8)	**<0.001**
Albumin (g/L)	39.0 (36.0-41.7)	40.3 (37.9-42.7)	**<0.001**	39.1 (36.4-41.6)	40.0 (37.7-42.2)	**<0.001**	38.9 (35.5-41.9)	40.7 (38.2-43.1)	**<0.001**
Platelet count (10 ^9^ /L)	245.0 (201.0-296.0)	254.0 (213.0-301.0)	**<0.001**	253.0 (210.0-303.0)	266.0 (225.0-313.0)	**<0.001**	235.0 (191.0-287.0)	240.0 (202.0-286.0)	**<0.001**
Elevated glucose (%)	13824 (14.2)	11364 (28.8)	**<0.001**	5195 (10.3)	5801 (27.6)	**<0.001**	8629 (18.5)	5563 (30.2)	**<0.001**
Elevated BMI (%)	41684 (42.8)	32944 (83.5)	**<0.001**	18326 (36.2)	16896 (80.4)	**<0.001**	23358 (50.0)	16048 (87.2)	**<0.001**
Elevated triglycerides (%)	21240 (21.8)	18164 (46.1)	**<0.001**	10068 (19.9)	9160 (43.6)	**<0.001**	11172 (23.9)	9004 (48.9)	**<0.001**
Low HDL-C (%)	41670 (42.8)	22944 (58.3)	**<0.001**	23593 (46.6)	14357 (68.3)	**<0.001**	18077 (38.7)	8587 (46.6)	**<0.001**
Elevated BP (%)	47498 (48.8)	26627 (67.5)	**<0.001**	20187 (39.8)	13636 (64.9)	**<0.001**	27311 (58.5)	12991 (70.5)	**<0.001**
Cigarette smoke (%)			**<0.001**			0.554			**<0.001**
Current	9163 (9.4)	2210 (5.6)		92 (0.2)	44 (0.2)		9071 (19.4)	2166 (11.8)	
Former	5961 (6.1)	1288 (3.3)		56 (0.1)	19 (0.1)		5905 (12.6)	1269 (6.9)	
Never	82261 (84.5)	35933 (91.1)		50525 (99.7)	20954 (99.7)		31736 (67.9)	14979 (81.3)	
T2DM (%)	9970 (10.2)	8146 (20.7)	**<0.001**	3279 (6.5)	4138 (19.7)	**<0.001**	6691 (14.3)	4008 (21.8)	**<0.001**
CKD (%)	6582 (6.8)	1657 (4.2)	**<0.001**	2068 (4.1)	517 (2.5)	**<0.001**	4514 (9.7)	1140 (6.2)	**<0.001**
CVD (%)	10769 (11.1)	6616 (16.8)	**<0.001**	3023 (6.0)	2817 (13.4)	**<0.001**	7746 (16.6)	3799 (20.6)	**<0.001**
Current drinking alcohol (%)	14158 (14.5)	0 (0.0)	**<0.001**	290 (0.6)	0 (0.0)	**<0.001**	13868 (29.7)	0 (0.0)	**<0.001**
FIB4 (%)			**<0.001**			**<0.001**			**<0.001**
<1.3	51881 (61.2)	21327 (64.9)		30102 (67.8)	11619 (65.5)		21779 (54.0)	9708 (64.2)	
1.3-2.67	25572 (30.2)	10137 (30.9)		11596 (26.1)	5444 (30.7)		13976 (34.6)	4693 (31.1)	
> 2.67	7309 (8.6)	1387 (4.2)		2700 (6.1)	674 (3.8)		4609 (11.4)	713 (4.7)	

Baseline characteristics are expressed as frequencies (proportions) for categorical variables and as medians (interquartile ranges [IQRs]) for continuous variables.

The Mann-Whitney U test and the chi-square test were used to evaluate the differences in the distribution of continuous variables and categorical variables between groups, respectively. Missing data for ALT, AST, GGT (1.3% each), PLT (13%), and albumin (1.3%) due to incomplete medical records; treated as NA in the analysis.

MASLD, Metabolic dysfunction-associated steatotic liver disease; SBP, Systolic blood pressure; DBP, Diastolic blood pressure; BMI, Body mass index; AST, Aspartate aminotransferase; ALT, Alanine aminotransferase; GGT, Gamma-glutamyl transferase; HDL-C, High-density lipoprotein cholesterol; Elevated BP, Elevated blood pressure; T2DM, Type 2 diabetes; CKD, Chronic kidney disease; CVD, Cardiovascular disease; FIB-4, Fibrosis-4 index.

### 3.2 Association between changes in age and prevalence of MASLD by sex

MASLD prevalence increased progressively from young adulthood to old age, exhibiting distinct sex-specific trajectories. As shown in **Figure 2**; **Table 2** and **Supplementary Table S4**, MASLD prevalence was higher in males than in females among the younger population (18–50 years). However, after age 51, the prevalence in females surpasses that in males, with a peak prevalence of 45.2% observed at age 63 among women (*P* < 0.001). **Figure 2** and **Table 3** show age- and sex-stratified analyses by calendar year.

**Figure 2:**
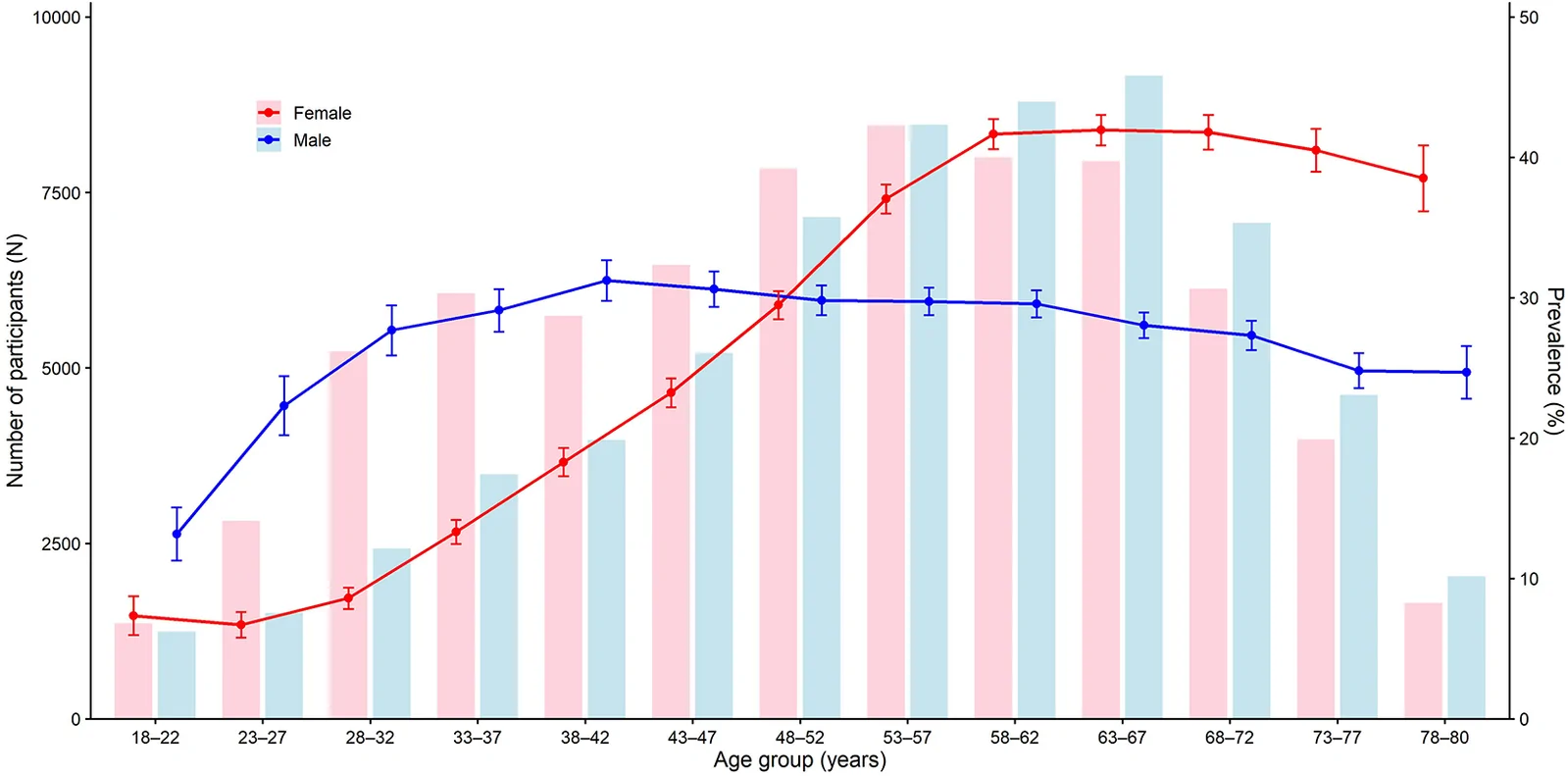
Trends in MASLD prevalence by age (aged 18–80).Bars represent number of participants (left y-axis); lines represent prevalence (%) with 95% confidence intervals (right y-axis). P for trend: male = 0.986, female <0.001.

**Table 2:** Key age-specific prevalence of MASLD across the study period (2014–2023).

Age (years)	Total N	Overall prevalence, %	Male prevalence, %	Female prevalence, %	*P* value for sex difference^a^
18	536	9.1	11.7	6.1	0.026
30	1545	13.1	25.9	7.4	<0.001
48	2723	27.9	30.8	25.4	0.002
49	2859	28.4	29.6	27.3	0.178
50	2893	29.5	31.3	27.9	0.045
51	3187	30.2	29.3	31.1	0.245
52	3321	31.7	28.6	34.7	<0.001
60	3289	34.4	28.2	41.1	<0.001
63 (Peak for Female)	3455	35.3	26.7	45.2	<0.001
80	1080	30.7	23.6	39.2	<0.001
*P* Value for trend ^b^		<0.001	0.986	<0.001	—

^a^ Statistical significance of differences in prevalence between males and females across different age groups. ^b^Overall, male and female prevalence rates were treated as continuous variables to calculate the linear trend from age 18 to 80 years.

**Table 3:** Annual Prevalence of MASLD in the Overall Population (2014–2023).

Year	Total N	Total cases	Overall prevalence, %	Male prevalence, %	Female prevalence, %	*P* value for sex difference^a^	OR	95% CI
2014	8802	2633	29.9	28.3	31.7	<0.001	1.19	1.08-1.30
2015	10429	2923	28.0	26.5	29.4	0.001	1.15	1.06-1.26
2016	12172	3574	29.4	27.2	31.1	<0.001	1.21	1.12-1.31
2017	13770	3737	27.1	26.1	28.0	0.010	1.11	1.02-1.20
2018	14347	3910	27.3	26.8	27.7	0.210	1.05	0.97-1.13
2019	15454	4187	27.1	26.9	27.3	0.583	1.02	0.95-1.10
2020	13567	3647	26.9	26.2	27.5	0.075	1.07	0.99-1.16
2021	21070	6553	31.1	31.9	30.3	0.010	0.93	0.87-0.98
2022	23478	7059	30.1	29.7	30.4	0.239	1.03	0.98-1.09
2023	3727	1208	32.4	32.7	32.2	0.748	0.98	0.85-1.12
*P* Value for trend ^b^			<0.001	<0.001	0.671	—	<0.001	

Data in the table are reported as an odds ratio (OR) with a 95% confidence interval (CI). ^a^Statistical significance of differences in prevalence between males and females across different years from 2014 to 2023. ^b^Overall, male and female prevalence rates were treated as continuous variables to calculate the linear trend over the 10-year period from 2014 to 2023.

All ORs for females versus males were derived from unadjusted binary logistic regression. Estimates are unadjusted. An OR > 1 indicates higher odds in females; an OR < 1 indicates higher odds in males.

In the overall population, females had higher odds of MASLD than males between 2014 and 2017 (**Table 3**). (OR > 1, P < 0.05). In the 18–50-year group (**Table 4**), females had a lower odds of MASLD than males, with ORs of 0.58 (95% CI: 0.48–0.69) in 2014 and 0.47 (95% CI: 0.36–0.61) in 2023. Conversely, in the 51–80-year group **(Table 5**), females had a higher odds of MASLD than males, with ORs of 1.71 (95% CI: 1.53–1.91) in 2014 and 1.36 (95% CI: 1.15–1.60) in 2023.

**Table 4:** Annual prevalence of MASLD in the 18–50 years age group (2014–2023).

Year	Total N	Total cases	Overall prevalence, %	Male prevalence, %	Female prevalence, %	*P* value for sex difference^a^	OR	95% CI
2014	3028	648	21.4	26.4	17.1	<0.001	0.58	0.48-0.69
2015	3926	764	19.5	25.4	15.8	<0.001	0.55	0.47-0.65
2016	4802	944	19.7	25.0	16.9	<0.001	0.61	0.53-0.70
2017	5485	1022	18.6	25.4	15.0	<0.001	0.52	0.45-0.59
2018	6029	1227	20.4	27.0	16.4	<0.001	0.52	0.47-0.60
2019	6347	1277	20.1	27.8	15.3	<0.001	0.47	0.41-0.53
2020	5639	1127	20.0	26.8	15.2	<0.001	0.49	0.43-0.56
2021	8129	1932	23.8	33.0	16.3	<0.001	0.40	0.36-0.44
2022	9318	2280	24.5	31.3	19.1	<0.001	0.52	0.47-0.57
2023	1308	323	24.7	31.8	17.9	<0.001	0.47	0.36-0.61
*P* Value for trend ^b^			<0.001	<0.001	0.004	—	0.001	

Data in the table are reported as an odds ratio (OR) with a 95% confidence interval (CI). ^a^Statistical significance of differences in prevalence between males and females across different years from 2014 to 2023. ^b^Overall, male and female prevalence rates were treated as continuous variables to calculate the linear trend over the 10-year period from 2014 to 2023.

All ORs for females versus males were derived from unadjusted binary logistic regression. Estimates are unadjusted. An OR > 1 indicates higher odds in females; an OR < 1 indicates higher odds in males.

**Table 5:** Annual prevalence of MASLD in the 51–80 years age group (2014–2023).

Year	Total N	Total cases	Overall prevalence, %	Male prevalence, %	Female prevalence, %	*P* value for sex difference^a^	OR	95% CI
2014	5774	1985	34.4	29.0	41.1	<0.001	1.71	1.53-1.91
2015	6503	2159	33.2	27.0	40.2	<0.001	1.82	1.64-2.02
2016	7370	2630	35.7	28.2	43.9	<0.001	2.00	1.81-2.19
2017	8285	2715	32.8	26.4	39.9	<0.001	1.86	1.69-2.04
2018	8318	2683	32.3	26.6	38.5	<0.001	1.73	1.57-1.89
2019	9107	2910	32.0	26.4	38.1	<0.001	1.72	1.57-1.88
2020	7928	2520	31.8	25.8	38.4	<0.001	1.79	1.63-1.97
2021	12941	4621	35.7	31.4	40.4	<0.001	1.48	1.38-1.59
2022	14160	4779	33.8	28.8	38.8	<0.001	1.57	1.46-1.68
2023	2419	885	36.6	33.1	40.2	<0.001	1.36	1.15-1.60
*P* Value for trend^b^			0.227	<0.001	0.002	—	<0.001	

Data in the table are reported as an odds ratio (OR) with a 95% confidence interval (CI). ^a^Statistical significance of differences in prevalence between males and females across different years from 2014 to 2023. ^b^Overall, male and female prevalence rates were treated as continuous variables to calculate the linear trend over the 10-year period from 2014 to 2023.

All ORs for females versus males were derived from unadjusted binary logistic regression. Estimates are unadjusted. An OR > 1 indicates higher odds in females; an OR < 1 indicates higher odds in males.

In the multivariable logistic regression model (**Table 6**), the sex × age interaction was statistically significant (*P* < 0.001), confirming that the sex difference in MASLD odds varied by age. After adjustment for calendar year, the interaction term remained significant. Because the model used males as the reference category, the main effect of sex represented the male-versus-female odds at age 50. To facilitate interpretation, we also calculated adjusted ORs for females versus males within each age group (**Table 7**). Women aged 18–50 years had lower adjusted odds of MASLD than men (aOR = 0.50, 95% CI: 0.48–0.52, P < 0.001), whereas women aged 51–80 years had higher adjusted odds (aOR = 1.69, 95% CI: 1.64–1.74, P < 0.001). These adjusted estimates were consistent with the unadjusted results. **Table 7** presents models adjusted only for calendar year, whereas **Supplementary Table S8** presents models further adjusted for BMI, glucose, triglycerides, HDL-C, T2DM, and CVD.

**Table 6:** Multivariable logistic regression for MASLD with sex, age, calendar year, and sex × age interaction.

Variable	B	S.E.	Wald	*P* value	OR	95% CI
Sex (female vs male)	0.096	0.013	53.901	<0.001	1.10	1.07–1.13
Age (per year, centered)	0.000	0.001	0.067	0.796	1.00	0.99–1.00
Calendar year (per year)	0.012	0.002	27.528	<0.001	1.01	1.01–1.02
Sex × age interaction	0.042	0.001	2316.086	<0.001	1.04	1.04–1.04

OR, odds ratio; CI, confidence interval. The model included sex, age (centered at 50 years), calendar year, and a sex × age interaction term. Sex was coded as male = 1, female = 0 (reference). Age was centered at 50 years.

**Table 7:** Adjusted odds ratios for MASLD by sex within age groups (female vs. male).

Age group	Female vs. male, adjusted OR (95% CI)	*P* value
18–50 years	0.50 (0.48–0.52)	<0.001
51–80 years	1.69 (1.64–1.74)	<0.001

Note: Adjusted ORs were derived from logistic regression models stratified by age group, adjusted for calendar year. An OR > 1 indicates higher odds in females; an OR < 1 indicates higher odds in males.

To further explore whether the sex difference was independent of metabolic risk factors, we performed additional multivariable logistic regression models stratified by age group, adjusting for age, BMI, glucose, triglycerides, HDL-C, T2DM, CVD, and calendar year (**Supplementary Table S8**). After full adjustment, the sex difference was no longer statistically significant in the 18–50 year age group (aOR = 0.974, 95% CI: 0.925–1.025, *P* = 0.311), whereas women aged 51–80 years had significantly higher odds of MASLD than men (aOR = 2.342, 95% CI: 2.262–2.426, *P* < 0.001).

Further analysis of the age-specific prevalence (**Table 2**) showed a significant linear increase with age in females (P for trend < 0.001) but not in males (P for trend = 0.986). In contrast, the temporal trend analysis (**Figure 3**) showed that MASLD prevalence increased significantly from 2014 to 2023 in males across all age strata, and in females within both the 18–50 year and 51–80 year groups (all P < 0.05; **Figures 3A–C**). However, Joinpoint regression analysis of male prevalence identified three joinpoints at ages 25, 34, and 59 years. Prevalence increased significantly from age 18 to 25 years (APC = 12.97%, 95% CI: 7.25–30.00, *P* = 0.006), plateaued from 25 to 59 years (APCs not statistically significant), and then declined significantly from 59 to 80 years (APC = −1.01%, 95% CI: −2.85 - −0.10, *P* = 0.048). The overall AAPC was 1.41% (95% CI: 1.05–1.89, P < 0.001; **Supplementary Table S5**; **Supplementary Figure S1**). Male MASLD prevalence thus exhibited a nonlinear age pattern: a steep increase from 18 to 25 years, a plateau from 25 to 59 years, and a significant decline from 59 to 80 years.

**Figure 3:**
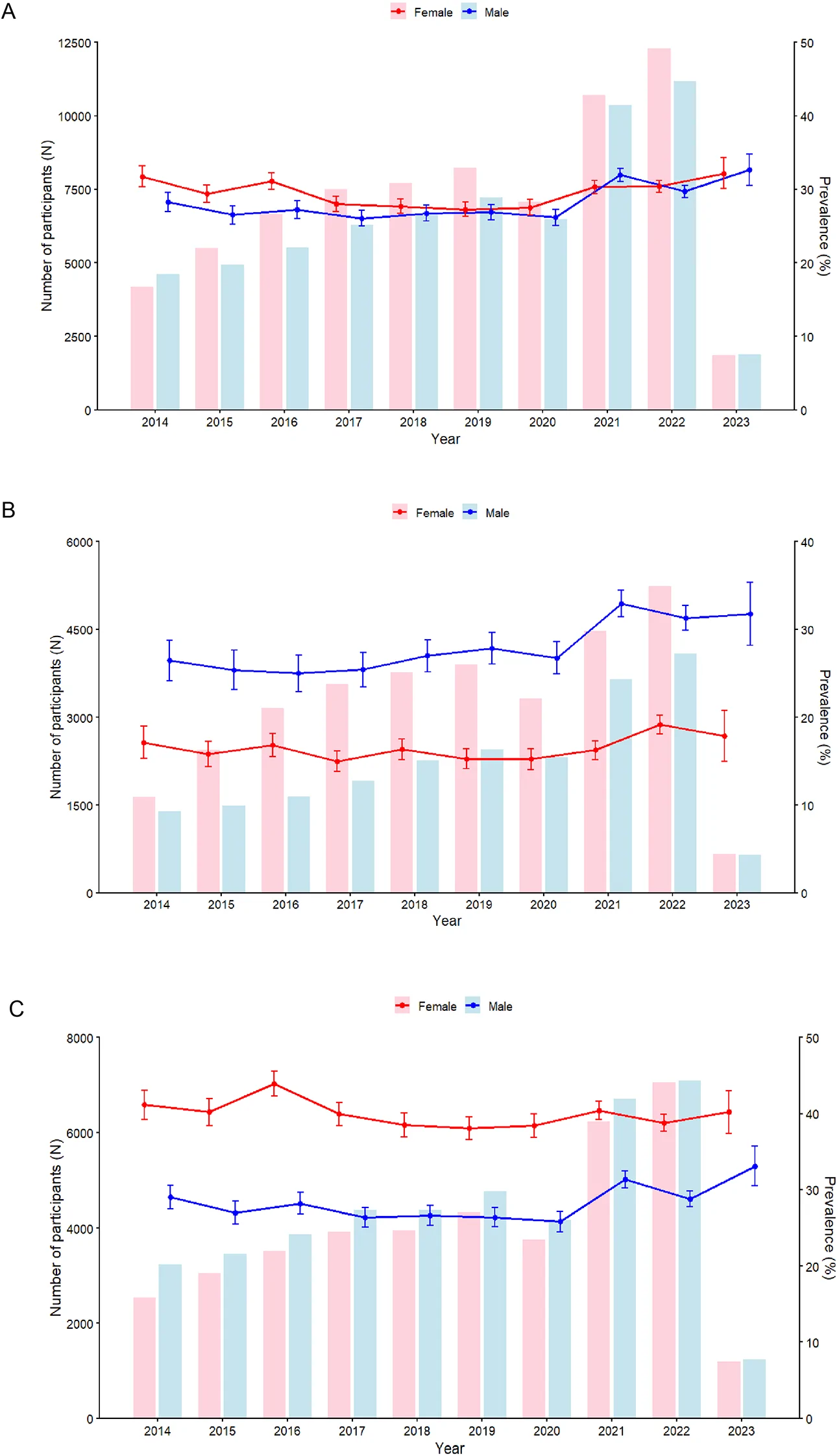
Temporal trends in MASLD prevalence stratified by sex (2014–2023). **(A)** individuals aged 18–80; **(B)** individuals aged 18–50; **(C)** Individuals aged 51–80. Bars represent number of participants (left y-axis); lines represent prevalence (%) with 95% confidence intervals (right y-axis). P for trend: **(A)** male <0.001, female = 0.671; **(B)** male <0.001, female=0.004; **(C)** male <0.001, female = 0.002.

### 3.3 Clinical characteristics of MASLD patients: an analysis by sex and age

Participants were categorized into two age groups: 18–50 years (young and middle-aged adults) and 51–80 years (older adults). To elucidate age- and sex-specific prevalence patterns, we performed comprehensive stratified analyses by sex and age group to identify key metabolic and clinical factors associated with MASLD within each stratum.

As shown in **Supplementary Tables S6, S7**, significant differences in metabolic parameters were observed between MASLD and non-MASLD participants across both age strata. Overall, MASLD patients exhibited a more adverse metabolic profile, including higher body mass index (BMI), elevated systolic and diastolic blood pressure (SBP/DBP), increased triglycerides (TG), and lower high-density lipoprotein cholesterol (HDL-C) (all *P* < 0.001). Overall, the prevalence of MASLD was highest among women aged 51–80 years.

Regarding fibrosis risk, the distribution of FIB-4 categories differed significantly by sex and age group (all P < 0.001; **Supplementary Table S7**). Among women with MASLD, the proportion with FIB-4 >2.67 was higher in the 51–80 year group than in the 18–50 year group (4.6% vs. 1.4%). A similar pattern was observed in men with MASLD (6.4% vs. 1.5%). These findings indicate that advanced fibrosis risk, as estimated by FIB-4, increases with age in both sexes, with older women with MASLD representing a high-risk subgroup.

## 4 Discussion

The findings of this large single-center cross-sectional study demonstrate a sex-specific reversal of MASLD prevalence with age: men under 50 years have a higher prevalence, whereas women over 50 years bear a greater metabolic burden and higher MASLD prevalence. Female participants showed an age-related increase in prevalence, surpassing that in men after age 51 and peaking at 63 years. This large-scale study provides a detailed analysis of the relationships between sex, age, and MASLD prevalence in a hospitalized Chinese patient group. Importantly, these findings remained robust after adjustment for age, calendar year, and their interaction. The significant sex × age interaction (*P* < 0.001) further supports the notion that the effect of sex on MASLD prevalence is age-dependent. In the adjusted model, women aged 18–50 years had approximately half the odds of MASLD compared with men, whereas women aged 51–80 years had 69% higher odds than men. These adjusted estimates were consistent with the unadjusted results, reinforcing the robustness of the observed associations.

However, when we further adjusted for metabolic risk factors (BMI, glucose, triglycerides, HDL-C, T2DM, and CVD) in age-stratified models, the sex difference in the younger age group was no longer statistically significant (aOR = 0.974, 95% CI: 0.925–1.025, *P* = 0.311), whereas the higher prevalence in older women became more pronounced (aOR = 2.342, 95% CI: 2.262–2.426, *P* < 0.001; **Supplementary Table S8**). This suggests that the lower MASLD prevalence observed in younger women are largely mediated by their more favorable metabolic profile, whereas the higher prevalence in older women are independent of traditional metabolic risk factors. These findings highlight the importance of considering age-specific mechanisms and suggest that factors beyond conventional metabolic parameters may contribute to the elevated MASLD prevalence in older women.

MASLD is a sexually dimorphic disorder, with its prevalence and severity increasing markedly in women aged ≥50 years (16). Perimenopausal endocrine changes typically begin around age 45, with the average menopausal age for Asian women (including Chinese women) being approximately 50 years (29). Early epidemiological surveys using previous NAFLD criteria also observed this pattern. For instance, a study of 4,009 administrative officials in Shanghai found that NAFLD prevalence was lower in premenopausal women than in men, but this trend reversed after age 50, with prevalence in women rising to levels comparable with men of the same age (30). Similarly, a cross-sectional study of 3,543 residents in Guangdong reported a higher prevalence in men under 50 but a higher prevalence in women over 50 (31). Even after adjusting for age and BMI, the elevated prevalence of MASLD in women aged ≥50 years remained significant (11–13). A recent study reported that the number of prevalent MASLD cases was highest in younger adults, peaking at ages 35–39 years in males and at ages 55–59 years in females (32). Overall, our study of hospitalized Chinese patients reinforces that the sex difference in MASLD prevalence is not uniform across ages but exhibits a distinct age-dependent pattern: men under 50 have a higher prevalence, whereas women over 50 bear a greater burden.

There was evidence suggesting that men under 50 years have a consistently higher MASLD prevalence than women. This sex difference during reproductive years is primarily attributed to the protective effects of estrogen in women aged <50 years (2, 7). In men, higher androgen levels promote visceral adiposity, which is metabolically more harmful than subcutaneous fat. Visceral fat accumulation increases free fatty acid flux to the liver, impairs insulin sensitivity, and promotes hepatic *de novo* lipogenesis (7). In addition, men exhibit lower adiponectin levels and higher pro-inflammatory cytokine profiles (e.g., TNF-α, IL-6) compared with women aged <50 years, further exacerbating hepatic steatosis and inflammation (7). Lifestyle factors, including higher rates of alcohol consumption and less favorable dietary patterns in younger men, may also contribute, although our study assessed alcohol only as current drinking status and did not exclude participants based on alcohol use. Furthermore, animal models have demonstrated that male rodents develop more severe steatosis and steatohepatitis than females when fed a high-fat diet, supporting the role of sex hormones in modulating hepatic lipid metabolism (33, 34). Accordingly, age- and sex-specific discrepancies should be fully considered when designing population-level intervention strategies. In the hospitalized setting, these differences may be even more pronounced, as younger men with comorbidities are more likely to undergo evaluation. Therefore, Targeted interventions tailored to distinct age and sex subgroups are essential to curb the rising prevalence of MASLD.

Our study demonstrates that hospitalized women aged ≥50 years bear a higher metabolic burden and MASLD prevalence, consistent with observations in dysmetabolic groups where older women with steatotic liver disease exhibit worse metabolic profiles (35). This is manifested as elevated BMI, increased SBP and DBP, increased triglycerides (TG), and lower high-density lipoprotein cholesterol (HDL-C), often accompanied by T2DM. These findings are consistent with the hypothesis that age-related hormonal changes may contribute to the observed patterns; however, direct hormone data were not available, and this mechanism remains speculative. Although MASLD occurs across all age groups, the prevalence rises markedly in women aged ≥50 years (9, 10). Consistently, a study of patients with endometrial cancer showed that earlier surgical menopause (particularly at age <50 years) significantly increases the odds of NAFLD (36). The mechanisms underlying these sex differences remain incompletely understood; estrogen-related pathways have been proposed but could not be directly assessed in this study (16, 37–39). However, evidence from studies on hormone replacement therapy (HRT) and MASLD risk has been inconsistent. While some observational studies suggest a protective effect of HRT, others—including prospective cohorts and nested case–control analyses—found no significant reduction in MASLD prevalence, and some reported even higher MASLD risk (40, 41). Preliminary studies have confirmed sex differences in the occurrence, diagnosis, and clinical presentation of MASLD in women (35, 42, 43), and that the prevalence and disease progression of MASLD may accelerate in women aged ≥50 years (44). A recent prospective study further demonstrated that genetic predisposition, particularly the PNPLA3 G/G genotype, significantly modifies the odds of age-associated increases in fibrosis in patients with MASLD (45). A recent trajectory analysis of comorbidity in MASLD patients revealed not only an elevated odds of multiple coexisting conditions but also a distinct temporal sequence, typically progressing from metabolic disorders to cardiovascular diseases and ultimately to multi-system complications (46). Consistent with the age-related increase in MASLD prevalence, the proportion of patients with elevated FIB-4 (>2.67) was substantially higher in older women with MASLD than in younger women (**Supplementary Table S7**). This suggests that older women with MASLD may carry a higher burden of advanced fibrosis and should be prioritized for fibrosis risk stratification and surveillance. Consequently, systematic screening (e.g., annual liver ultrasound) and comprehensive metabolic management should be prioritized for women aged ≥50 years with additional odds factors such as obesity or diabetes.

Joinpoint regression analysis revealed a nonlinear age trend in men: prevalence rose steeply from 18 to 25 years, plateaued until 59 years, and then declined significantly thereafter. This pattern contrasts with the sustained higher prevalence in women after age 50, with a crossover of sex-specific prevalence observed. One possible explanation is that survival selection bias, men who survive to older ages may represent a healthier subset, as men with severe MASLD and its complications (e.g., cardiovascular disease, advanced fibrosis, cirrhosis, hepatocellular carcinoma) have higher mortality rates (7, 45). In addition, the age-standardized DALY rate for MASLD is higher in men than in women in many populations, suggesting that men experience greater disease severity and die earlier (7); therefore, the declining prevalence in older men might partly reflect differential mortality. In addition, the proportion of men with FIB-4 >2.67 also increased with age (6.4% in 51–80 years vs. 1.5% in 18–50 years; **Supplementary Table S7**), indicating that older men who survive with MASLD may still carry a substantial burden of advanced fibrosis. This is consistent with the higher MASLD-related mortality observed in men. Furthermore, it is also plausible that age-related physiological changes in men may alter metabolic odds. Although total testosterone declines with aging, the ratio of estrogen to androgen may shift in a direction that modestly attenuates hepatic fat accumulation. However, some studies have shown that hypogonadal men are closely associated with alterations in body composition (e.g., decreased muscle mass and increased fat mass) as well as the development of insulin resistance, although the net effect on hepatic steatosis remains complex and requires further investigation (47). Finally, it is possible that older hospitalized men in our study were born in earlier decades when obesity and metabolic syndrome were less prevalent. Consequently, secular trends in lifestyle and dietary habits may have resulted in lower lifetime exposure to metabolic odds factors in the oldest age groups.

The odds ratios reported in **Table 3**-**5** were derived from unadjusted logistic regression models and should therefore be interpreted as crude associations rather than independent effects. Although we subsequently performed multivariable logistic regression adjusted for age, BMI, glucose, triglycerides, HDL-C, T2DM, CVD, and calendar year, several potential confounders could not be fully accounted for, including dietary patterns, physical activity, socioeconomic status, waist circumference, HbA1c, medication use, and menopausal status. Although the direction and significance of the sex × age interaction remained consistent after multivariable adjustment, residual confounding by unmeasured factors cannot be entirely excluded.

These findings carry clear implications for clinical practice and public health policy. In this hospitalized population, targeted screening and lifestyle intervention should be implemented for men under 50 years, particularly those with obesity or metabolic syndrome. For hospitalized women aged ≥50 years, especially those with obesity, type 2 diabetes, or hypertension, systematic screening and comprehensive metabolic management should be prioritized. Health education campaigns should raise awareness of MASLD as a common but underrecognized condition. Lifestyle modifications remain the cornerstone of management: weight loss of 5–10% significantly reduces hepatic steatosis and may reverse fibrosis. Structured programs, including dietary counseling and physical activity, should be offered. Comorbidity management is essential, as aggressive control of blood pressure, blood glucose, and lipids reduces cardiovascular risk—the leading cause of death in MASLD patients. Finally, surveillance for advanced liver disease (cirrhosis and hepatocellular carcinoma) should be considered in older men and women with evidence of significant fibrosis (FIB-4 > 2.67) (45). A recent global burden study estimated that 1.3 billion people had MASLD in 2023, with numbers projected to reach 1.8 billion by 2050, underscoring the urgent need for the implementation of these strategies (32).

## 5 Limitations

Several limitations of this study should be acknowledged. First, as a single-center study conducted in a hospitalized population undergoing liver ultrasonography at a tertiary hospital, the findings may not be generalizable to the general population or community settings. Second, the cross-sectional design limits causal inference regarding the effects of age and sex on MASLD, and reliance on ultrasound-based data without histological confirmation may lead to an overestimation of MASLD prevalence. Third, missing data were substantial: of the initial 255,187 patients, 118,371 (46.4%) were excluded due to missing values required for MASLD classification. This high exclusion rate is a recognized limitation. Nevertheless, the included and excluded groups were broadly comparable, suggesting that clinically meaningful selection bias is unlikely. For the remaining variables (ALT/AST/GGT, platelet count, and albumin), missing rates were low, and we did not perform imputation. The primary reasons for missing data were administrative (incomplete electronic medical records at earlier time points) rather than biological, which may partially mitigate bias, but we cannot rule out residual confounding entirely. Additional details on the comparability of the two groups are provided in **Supplementary Tables S1**–**S3**. Fourth, several key parameters recommended by the Delphi consensus criteria for MASLD diagnosis—including waist circumference and HbA1c—were not available in our dataset. Their absence may have led to underdiagnosis or misclassification of some cases (both waist circumference and HbA1c were unavailable). Additionally, data on antihypertensive and lipid-lowering therapy were unavailable, as were detailed quantitative data on alcohol consumption. Fifth, the associations between MASLD prevalence and factors such as dietary patterns, physical activity, fructose intake, or environmental exposure to toxic metals were not evaluated. Sixth, the lack of molecular biology and metabolomics data hinders a comprehensive understanding of disease progression and clinical features of MASLD in the elderly population. The study does not provide mechanistic insights into the observed sex differences. Future studies with direct hormone measurements are needed to test the hypothesis that age-related hormonal changes contribute to the higher MASLD prevalence in older women. Finally, there may be unmeasured confounders. Particularly factors not captured by routine health screenings, such as stress or other mental health factors.

## 6 Conclusion

In this single-center cross-sectional study of hospitalized patients undergoing liver ultrasonography, MASLD prevalence exhibited a clear age-dependent sex reversal. Men aged <50 years have a higher prevalence and represent a key target for early lifestyle intervention, with Joinpoint regression confirming a nonlinear age pattern in men: a steep increase in young adulthood, a plateau in middle age, and a significant decline in older age. Women aged ≥50 years with obesity, type 2 diabetes, or hypertension, constitute another high-odds group requiring systematic screening and metabolic management. These findings support age- and sex-specific screening in hospital-based clinical practice, including education on diet and physical activity, weight reduction, and surveillance for advanced liver disease in those with elevated FIB-4.

## Data Availability

The data analyzed in this study is subject to the following licenses/restrictions: The datasets generated and analyzed during the present study are available from the corresponding author upon reasonable request. Requests to access these datasets should be directed to Suosu Wei sswei@gxams.org.cn.
