# Supplementary material for: Sex differences in the prevalence of metabolic dysfunction-associated steatotic liver disease in hospitalized patients: a single-center cross-sectional study
Source: Front Endocrinol (Lausanne). 2026 Oct 22;17:1942008. doi: 10.3389/fendo.2026.1942008 (PMC13735668; doi:10.3389/fendo.2026.1942008)
Supplement: Supplementary file 1 [file Supplementaryfile1.docx]

**SUPPLEMENTARY MATERIALS**

**Supplementary Figure**

**Supplementary Figure S1.** Joinpoint regression analysis of crude MASLD prevalence in males by age (18–80 years).(Page 2)

**Supplementary Tables**

**Supplementary Table S1**. Distribution of Excluded Participants by Calendar Year(Page 3)

**Supplementary Table S2**. Demographic Characteristics of Excluded Participants by Calendar Year(Page 3)

**Supplementary Table S3**. Baseline Characteristics of Included and Excluded Groups(Page 4)

**Supplementary Table S4**. Complete Age-Specific Prevalence of MASLD (18–80 Years)	(Page 6)

**Supplementary Table S5.** Joinpoint regression analysis of male MASLD prevalence by age(Page 9)

**Supplementary Table S6.** Characteristics of patients with MASLD stratified by age

(Page 10)

**Supplementary Table S7.** Characteristics of MASLD patients according to sex and age stratification	(Page 12)

**Supplementary Table S8.** Multivariable logistic regression for MASLD stratified by age group (Page 16)

**Supplementary Figure S1.** **Joinpoint regression analysis of crude MASLD prevalence in males by age (18–80 years). Red squares represent observed crude prevalence (per 100,000). Colored lines represent fitted Joinpoint segments. Joinpoints were identified at ages 25, 34, and 59 years. The APC for each segment is shown in the legend. * indicates APC significantly different from zero at α = 0.05. The overall AAPC was 1.41% (95% CI: 1.05–1.89, P < 0.001). Detailed APC values are provided in Supplementary Table S5.**

**Supplementary Table S1. Distribution of Excluded Participants by Calendar Year**

| **Year** | **Total Initial Participants (N)** | **Excluded Participants (n)** | **Exclusion Rate (%)** | ***P*-value** | ***P* for trend** |
| --- | --- | --- | --- | --- | --- |
| **2014** | **20011** | **11209** | **56.0** | **<0.001** | **<0.001** |
| **2015** | **21265** | **10836** | **51.0** |  |  |
| **2016** | **22415** | **10243** | **45.7** |  |  |
| **2017** | **22751** | **8981** | **39.5** |  |  |
| **2018** | **23413** | **9066** | **38.7** |  |  |
| **2019** | **26085** | **10631** | **40.8** |  |  |
| **2020** | **24608** | **11041** | **44.9** |  |  |
| **2021** | **30566** | **9496** | **31.1** |  |  |
| **2022** | **35898** | **12420** | **34.6** |  |  |
| **2023** | **28175** | **24448** | **86.8** |  |  |

***P*-value** derived from the chi-square test comparing the distribution of included vs. excluded participants across calendar years. A non-significant P-value (> 0.05) indicates that exclusion rates did not vary significantly across years.

***P* for trend** derived from the Cochran-Armitage trend test to assess whether exclusion rates changed monotonically over the 10-year study period. A non-significant P for trend (> 0.05) indicates no systematic increase or decrease in exclusion rates over time.

**Table Note:** Exclusion rates were calculated as the number of excluded participants divided by the total initial participants for each calendar year. The distribution of excluded participants across years was generally balanced, with no evidence of concentration in any particular period. The chi-square test P-value for year-to-year differences was > 0.05, and the *P* for trend was > 0.05, indicating that exclusion rates did not significantly change over the study period.

**Supplementary Table S2. Demographic Characteristics of Excluded Participants by Calendar Year**

| **Year** | **Sex Distribution of Excluded Participants** | | | | **Age Group Distribution of Excluded Participants** | | | |
| --- | --- | --- | --- | --- | --- | --- | --- | --- |
|  | **Male**  **n (%)** | **Female**  **n (%)** | **P-value** | ***P* for trend** | **18–50 years**  **n (%)** | **51–80 years**  **n (%)** | ***P*-value** | ***P* for trend** |
| 2014 | 4384(39.1) | 6825(60.9) | **<0.001** | **<0.001** | 6233(55.6) | 4976(44.4) | **<0.001** | **<0.001** |
| 2015 | 4509(41.6) | 6327(58.4) |  |  | 5942(54.8) | 4894(45.2) |  |  |
| 2016 | 4383(42.8) | 5860(57.2) |  |  | 5488(53.6) | 4755(46.4) |  |  |
| 2017 | 3793(42.2) | 5188(57.8) |  |  | 4788(53.3) | 4193(46.7) |  |  |
| 2018 | 3667(40.4) | 5399(59.6) |  |  | 5127(56.6) | 3939(43.4) |  |  |
| 2019 | 3533(33.2) | 7098(66.8) |  |  | 6722(63.2) | 3909(36.8) |  |  |
| 2020 | 3472(31.4) | 7569(68.6) |  |  | 7120(64.5) | 3921(35.5) |  |  |
| 2021 | 3544(37.3) | 5952(62.7) |  |  | 5644(59.4) | 3852(40.6) |  |  |
| 2022 | 4727(38.1) | 7693(61.9) |  |  | 7277(58.6) | 5143(41.4) |  |  |
| 2023 | 11113(45.5) | 13335(54.5) |  |  | 10792(44.1) | 13656(55.9) |  |  |

*P*-values for the distribution of sex and age groups across calendar years were derived from the chi-square test.

*P* for trend was derived from the Cochran-Armitage trend test to assess whether the proportions changed monotonically over time. A non-significant *P*-value (> 0.05) for both tests indicates that the sex and age compositions of excluded participants did not vary significantly across years and showed no systematic change over the study period.

Table Note: The sex and age distributions of excluded participants remained stable across years, with all *P*-values > 0.05. These findings suggest that missingness was not systematically biased toward any specific demographic subgroup over the study period.

**Supplementary Table S3. Baseline Characteristics of Included and Excluded Groups**

| **Variables** | **Included Group (n=136,816)** | **Excluded Group (n=118,371)** | ***P*-value** | **Absolute SMD** |
| --- | --- | --- | --- | --- |
| **Age (years)** | 55.0(43.0-65.0) | 48.0(34.0-61.0) | **<0.001** | **0.35** |
| **Male(%)** | 65126(47.6) | 47125(39.8) | **<0.001** | **0.16** |
| **SBP (mmHg)** | 129.0(116.0-145.0) | 123.0(111.0-138.0) | **<0.001** | **0.00** |
| **DBP (mmHg)** | 78.0(70.0-87.0) | 76.0(68.0-84.0) | **<0.001** | **0.06** |
| **BMI (kg/m^2^)** | 23.4(21.1-25.8) | 23.2(20.8-25.8) | **<0.001** | **0.03** |
| **AST (IU/L)** | 20.1(17.0-26.0) | 20.0(16.0-25.0) | **<0.001** | **0.01** |
| **ALT (IU/L)** | 16.9(12.0-25.0) | 15.0(10.5-24.0) | **<0.001** | **0.00** |
| **GGT (IU/L)** | 24.0(16.0-40.0) | 21.0(14.0-36.0) | **<0.001** | **0.04** |
| **Triglycerides (mmol/L)** | 1.2(0.9-1.8) | 1.3(0.9-1.8) | **<0.001** | **0.02** |
| **HDL-C (mmol/L)** | 1.2(0.9-1.4) | 1.2(0.9-1.4) | **<0.001** | **0.03** |
| **glucose (mmol/L)** | 4.7(4.3-5.3) | 4.8(4.4-5.4) | **<0.001** | **0.11** |
| **Albumin (g/L)** | 39.4(36.6-42.0) | 39.4(37.0-42.0) | **<0.001** | **0.05** |
| **Platelet count (10^9^/L)** | 248.0(204.0-298.0) | 248.0(205.0-297.0) | **<0.001** | **0.00** |
| **Elevated glucose (%)** | 25188(18.4) | 8883(16.5) | **<0.001** | **0.05** |
| **Elevated BMI (%)** | 74628(54.5) | 63905(54.0) | **<0.001** | **0.01** |
| **Elevated triglycerides (%)** | 39404(28.8) | 21778(37.6) | **<0.001** | **0.19** |
| **Low HDL-C (%)** | 25634(18.7) | 9848(10.2) | **<0.001** | **0.24** |
| **Elevated BP (%)** | 74125(54.2) | 40416(43.4) | **<0.001** | **0.22** |
| **Cigarette smoke (%)** |  |  | **<0.001** |  |
| **Current** | 11373(8.3) | 5514(4.7) |  | **0.15** |
| **Former** | 7249(5.3) | 3834(3.2) |  | **0.10** |
| **Never** | 118194(86.4) | 109023(92.1) |  | **0.18** |
| **T2DM (%)** | 18116(13.2) | 12273(10.4) | **<0.001** | **0.09** |
| **CKD (%)** | 8239(6.0) | 5499(4.6) | **<0.001** | **0.06** |
| **CVD (%)** | 17385(12.7) | 7488(6.3) | **<0.001** | **0.22** |
| **Current drinking alcohol**  **(%)** | 14158(10.3) | 7070(6.0) | **<0.001** | **0.16** |
| **FIB4(%)** |  |  | **<0.001** |  |
| **<1.3** | 73208(62.2) | 71791(69.5) |  | **0.15** |
| **1.3-2.67** | 35709(30.4) | 24763(24.0) |  | **0.14** |
| **＞2.67** | 8689(7.4) | 6687(6.5) |  | **0.04** |

**Abbreviations:** MASLD, Metabolic dysfunction-associated steatotic liver disease; SMD, Standardized Difference; SBP, Systolic blood pressure; DBP, Diastolic blood pressure; BMI, Body mass index; AST, Aspartate aminotransferase; ALT, Alanine aminotransferase; GGT, Gamma-glutamyl transferase; HDL-C, High-density lipoprotein cholesterol; Elevated BP, Elevated blood pressure; T2DM, Type 2 diabetes; CKD, Chronic kidney disease; CVD, Cardiovascular disease; FIB-4, Fibrosis-4 index.

***P* values** were derived from Mann-Whitney U test for continuous variables (presented as median [IQR]) and chi-square test for categorical variables (presented as n [%]).

**Absolute SMD** was calculated as: for continuous variables, |(Mean₁ – Mean₂) / √((SD₁² + SD₂²)/2)|; for categorical variables, |(p₁ – p₂) / √((p₁(1–p₁)+p₂(1–p₂))/2)|. An absolute SMD < 0.1 indicates negligible imbalance; < 0.2 is generally considered acceptable for baseline comparability.

**Supplementary Table S4.** **Complete Age-Specific Prevalence of MASLD (18–80 Years)**

| **Age (years)** | **Total N** | **Overall Prevalence, %** | **Male Prevalence, %** | **Female Prevalence, %** | ***P* Value for Sex Difference** |
| --- | --- | --- | --- | --- | --- |
| 18 | 536 | 9.1 | 11.7 | 6.1 | **0.026** |
| 19 | 488 | 9.0 | 8.2 | 10.0 | 0.474 |
| 20 | 466 | 7.7 | 10.0 | 5.7 | 0.077 |
| 21 | 535 | 11.0 | 15.0 | 7.8 | **0.008** |
| 22 | 582 | 13.1 | 22.1 | 7.3 | **<0.001** |
| 23 | 652 | 9.5 | 16.2 | 5.1 | **<0.001** |
| 24 | 731 | 11.9 | 21.4 | 6.3 | **<0.001** |
| 25 | 865 | 13.3 | 22.5 | 8.1 | **<0.001** |
| 26 | 941 | 13.2 | 25.9 | 6.6 | **<0.001** |
| 27 | 1137 | 12.0 | 24.1 | 6.8 | **<0.001** |
| 28 | 1327 | 12.7 | 23.7 | 7.7 | **<0.001** |
| 29 | 1402 | 14.8 | 29.6 | 8.8 | **<0.001** |
| 30 | 1545 | 13.1 | 25.9 | 7.4 | **<0.001** |
| 31 | 1615 | 14.9 | 28.3 | 8.2 | **<0.001** |
| 32 | 1778 | 17.1 | 30.0 | 10.5 | **<0.001** |
| 33 | 1844 | 16.1 | 27.0 | 10.8 | **<0.001** |
| 34 | 1964 | 19.5 | 31.0 | 13.4 | **<0.001** |
| 35 | 1923 | 19.3 | 29.7 | 13.5 | **<0.001** |
| 36 | 1931 | 20.4 | 30.8 | 13.8 | **<0.001** |
| 37 | 1885 | 20.1 | 26.9 | 15.4 | **<0.001** |
| 38 | 1863 | 23.2 | 32.0 | 17.7 | **<0.001** |
| 39 | 1864 | 23.2 | 32.4 | 17.1 | **<0.001** |
| 40 | 1973 | 23.3 | 29.5 | 18.9 | **<0.001** |
| 41 | 1989 | 22.9 | 31.3 | 17.2 | **<0.001** |
| 42 | 2024 | 25.1 | 31.2 | 20.4 | **<0.001** |
| 43 | 2295 | 24.1 | 29.9 | 19.7 | **<0.001** |
| 44 | 2190 | 25.8 | 30.5 | 22.2 | **<0.001** |
| 45 | 2261 | 26.6 | 30.8 | 23.1 | **<0.001** |
| 46 | 2462 | 27.6 | 30.5 | 25.2 | **0.003** |
| 47 | 2468 | 28.2 | 31.3 | 25.7 | **0.002** |
| 48 | 2723 | 27.9 | 30.8 | 25.4 | **0.002** |
| 49 | 2859 | 28.4 | 29.6 | 27.3 | 0.178 |
| 50 | 2893 | 29.5 | 31.3 | 27.9 | **0.045** |
| 51 | 3187 | 30.2 | 29.3 | 31.1 | 0.245 |
| 52 | 3321 | 31.7 | 28.6 | 34.7 | **<0.001** |
| 53 | 3408 | 31.5 | 28.0 | 35.1 | **<0.001** |
| 54 | 3436 | 33.1 | 29.4 | 36.9 | **<0.001** |
| 55 | 3310 | 33.1 | 30.5 | 35.8 | **0.001** |
| 56 | 3363 | 33.7 | 29.9 | 37.4 | **<0.001** |
| 57 | 3405 | 35.4 | 30.9 | 40.0 | **<0.001** |
| 58 | 3428 | 35.1 | 30.8 | 39.7 | **<0.001** |
| 59 | 3424 | 36.4 | 32.4 | 40.5 | **<0.001** |
| 60 | 3289 | 34.4 | 28.2 | 41.1 | **<0.001** |
| 61 | 3287 | 35.7 | 29.2 | 43.6 | **<0.001** |
| 62 | 3366 | 35.1 | 27.2 | 43.7 | **<0.001** |
| 63 | 3455 | 35.3 | 26.7 | 45.2 | **<0.001** |
| 64 | 3547 | 35.2 | 29.1 | 42.0 | **<0.001** |
| 65 | 3594 | 34.7 | 28.5 | 41.8 | **<0.001** |
| 66 | 3267 | 33.0 | 27.6 | 39.4 | **<0.001** |
| 67 | 3246 | 34.2 | 28.2 | 41.1 | **<0.001** |
| 68 | 3062 | 34.7 | 28.3 | 42.0 | **<0.001** |
| 69 | 2816 | 34.2 | 26.6 | 42.2 | **<0.001** |
| 70 | 2623 | 33.2 | 27.1 | 40.9 | **<0.001** |
| 71 | 2417 | 33.7 | 26.1 | 42.2 | **<0.001** |
| 72 | 2275 | 34.4 | 28.3 | 41.4 | **<0.001** |
| 73 | 2062 | 33.1 | 25.0 | 42.3 | **<0.001** |
| 74 | 1865 | 32.5 | 25.6 | 40.9 | **<0.001** |
| 75 | 1631 | 30.1 | 23.3 | 37.3 | **<0.001** |
| 76 | 1555 | 33.1 | 25.3 | 42.2 | **<0.001** |
| 77 | 1484 | 31.3 | 24.5 | 39.4 | **<0.001** |
| 78 | 1388 | 31.1 | 24.9 | 38.6 | **<0.001** |
| 79 | 1214 | 30.8 | 25.3 | 37.8 | **<0.001** |
| 80 | 1080 | 30.7 | 23.6 | 39.2 | **<0.001** |

**Supplementary Table S5. Joinpoint regression analysis of male MASLD prevalence by age**

| **Segment** | **Age range (years)** | **APC (95% CI)** | **AAPC (95% CI)** |
| --- | --- | --- | --- |
| 1 | 18–25 | 12.97% (7.25–30.00)* | — |
| 2 | 25–34 | 2.80% (−0.09–6.97) | — |
| 3 | 34–59 | −0.07% (−1.86–0.55) | — |
| 4 | 59–80 | −1.01% (−2.85–−0.10)* | — |
| Full range | 18–80 | — | 1.41% (1.05–1.89)* |

Note: APC, annual percent change; AAPC, average annual percent change; CI, confidence interval. * indicates statistical significance at α = 0.05.

Joinpoints were identified at ages 25, 34, and 59 years. The model was selected using the weighted Bayesian Information Criterion (WBIC)

**Supplementary Table S6** **Characteristics of patients with MASLD stratified by age**

| **Variables** | **18-50 years(n=54011)** | | ***P* value** | **51-80 years(n=82805)** | | ***P* value** |
| --- | --- | --- | --- | --- | --- | --- |
|  | **Non-MASLD**  **(n=42467)** | **MASLD**  **(n=11544)** |  | **Non-MASLD**  **(n=54918)** | **MASLD**  **(n=27887)** |  |
| **Age (years)** | 38.0(31.0-45.0) | 42.0(35.0-47.0) | 0.748 | 63.0(57.0-69.0) | 63.0(57.0-69.0) | **<0.001** |
| **Female(%)** | 26858(63.2) | 5299(45.9) | **<0.001** | 23815(43.4) | 15718(56.4) | **<0.001** |
| **SBP (mmHg)** | 119.0(108.0-131.0) | 128.0(117.0-140.0) | **<0.001** | 134.0(120.0-149.0) | 139.0(126.0-153.0) | **<0.001** |
| **DBP (mmHg)** | 75.0(68.0-84.0) | 82.0(75.0-90.0) | **<0.001** | 78.0(70.0-86.0) | 81.0(73.0-89.0) | **<0.001** |
| **BMI (kg/m^2^)** | 22.2(20.1-24.7) | 26.3(24.2-28.7) | **<0.001** | 22.5(20.5-24.7) | 25.4(23.6-27.4) | **<0.001** |
| **AST (IU/L)** | 19.0(16.0-24.0) | 21.0(17.0-27.0) | **<0.001** | 21.4(18.0-27.0) | 21.0(18.0-25.3) | **<0.001** |
| **ALT (IU/L)** | 14.0(10.0-23.0) | 22.3(15.0-36.0) | **<0.001** | 16.0(12.0-24.0) | 19.0(14.0-26.0) | **<0.001** |
| **GGT (IU/L)** | 18.0(13.0-31.0) | 31.0(21.0-53.0) | **<0.001** | 24.0(17.0-41.0) | 28.0(20.0-42.0) | **<0.001** |
| **Triglycerides (mmol/L)** | 1.1(0.8-1.6) | 1.7(1.2-2.5) | **<0.001** | 1.1(0.9-1.6) | 1.6(1.2-2.2) | **<0.001** |
| **HDL-C (mmol/L)** | 1.3(1.0-1.5) | 1.1(0.9-1.3) | **<0.001** | 1.2(1.0-1.4) | 1.1(1.0-1.3) | **<0.001** |
| **glucose (mmol/L)** | 4.5(4.1-4.9) | 4.8(4.4-5.4) | **<0.001** | 4.7(4.3-5.3) | 5.1(4.6-6.0) | **<0.001** |
| **Albumin (g/L)** | 39.9(37.0-42.6) | 41.4(39.0-43.8) | **<0.001** | 38.4(35.6-41.0) | 39.8(37.6-42.0) | **<0.001** |
| **Platelet count (10^9^/L)** | 253.0(210.0-303.0) | 274.0(229.0-323.0) | **<0.001** | 238.0(194.0-290.0) | 246.0(207.0-290.0) | **<0.001** |
| **Elevated glucose (%)** | 3921(9.2) | 2315(20.1) | **<0.001** | 9903(18.0) | 9049(32.4) | **<0.001** |
| **Elevated BMI (%)** | 17467(41.1) | 10142(87.9) | **<0.001** | 24217(44.1) | 22802(81.8) | **<0.001** |
| **Elevated triglycerides (%)** | 9825(23.1) | 5717(49.5) | **<0.001** | 11415(20.8) | 12447(44.6) | **<0.001** |
| **Low HDL-C (%)** | 17453(41.1) | 6727(58.3) | **<0.001** | 24217(44.1) | 16217(58.2) | **<0.001** |
| **Elevated BP (%)** | 14009(33.0) | 6499(56.3) | **<0.001** | 33489(61.0) | 20128(72.2) | **<0.001** |
| **Cigarette smoke (%)** |  |  | **<0.001** |  |  | **<0.001** |
| **Current** | 3070(7.2) | 695(6.0) |  | 6093(11.1) | 1515(5.4) |  |
| **Former** | 1205(2.8) | 233(2.0) |  | 4756(8.7) | 1055(3.8) |  |
| **Never** | 38192(89.9) | 10616(92.0) |  | 44069(80.2) | 25317(90.8) |  |
| **T2DM (%)** | 1648(3.9) | 1192(10.3) | **<0.001** | 8322(15.2) | 6954(24.9) | <**0.001** |
| **CKD (%)** | 2305(5.4) | 350(3.0) | **<0.001** | 4277(7.8) | 1307(4.7) | **<0.001** |
| **CVD (%)** | 1181(2.8) | 702(6.1) | **<0.001** | 9588(17.5) | 5914(21.2) | **<0.001** |
| **Current drinking alcohol**  **(%)** | 4513(10.6) | 0(0.0) | **<0.001** | 9545(17.6) | 0(0.0) | **<0.001** |
| **FIB4(%)** |  |  | **<0.001** |  |  | **<0.001** |
| **<1.3** | 32622(87.0) | 9031(92.4) |  | 19259(40.8) | 12296(53.3) |  |
| **1.3-2.67** | 3503(9.3) | 599(6.1) |  | 22069(46.7) | 9538(41.3) |  |
| **＞2.67** | 1380(3.7) | 143(1.5) |  | 5929(12.5) | 1244(5.4) |  |

Baseline characteristics are expressed as frequencies (proportions) for categorical variables and as medians (interquartile ranges [IQRs]) for continuous variables.

The Mann-Whitney U test and the chi-square test were used to evaluate the differences in the distribution of continuous variables and categorical variables between groups, respectively. Missing data for ALT, AST, GGT (1.3% each), PLT (13%), and albumin (1.3%) due to incomplete medical records; treated as NA in the analysis.

**Abbreviations:** MASLD, Metabolic dysfunction-associated steatotic liver disease; SBP, Systolic blood pressure; DBP, Diastolic blood pressure; BMI, Body mass index; AST, Aspartate aminotransferase; ALT, Alanine aminotransferase; GGT, Gamma-glutamyl transferase; HDL-C, High-density lipoprotein cholesterol; Elevated BP, Elevated blood pressure; T2DM, Type 2 diabetes; CKD, Chronic kidney disease; CVD, Cardiovascular disease; FIB-4, Fibrosis-4 index.

**Supplementary Table S7**  **Characteristics of MASLD patients according to sex and age stratification**

| **Variables** | **Female (n=71690)** | | | | | | **Male (n=65126)** | | | | | |
| --- | --- | --- | --- | --- | --- | --- | --- | --- | --- | --- | --- | --- |
|  | **18-50 years(n=32157)** | | ***P* value** | **51-80 years(n=39533)** | | ***P* value** | **18-50 years(n=21854)** | | ***P* value** | **51-80 years(n=43272)** | | ***P* value** |
|  | **Non-MASLD**  **(n=26858)** | **MASLD**  **(n=5299)** |  | **Non-MASLD**  **(n=23815)** | **MASLD**  **(n=15718)** |  | **Non-MASLD**  **(n=15609)** | **MASLD**  **(n=6245)** |  | **Non-MASLD**  **(n=31103)** | **MASLD**  **(n=12169)** |  |
| **Age (years)** | 37.0  (31.0-44.0) | 43.0  (36.0-47.0) | **<0.001** | 62.0  (56.0-69.0) | 63.0  (57.0-69.0) | **<0.001** | 40.0  (32.0-46.0) | 41.0  (35.0-47.0) | **<0.001** | 63.0  (57.0-70.0) | 63.0  (57.0-69.0) | **<0.001** |
| **SBP (mmHg)** | 115.0  (106.0-126.0) | 125.0  (114.0-137.0) | **<0.001** | 132.0  (118.0-147.0) | 139.0  (125.0-152.0) | **<0.001** | 125.0  (115.0-138.0) | 131.0  (121.0-142.0) | **<0.001** | 135.0  (121.0-150.0) | 140.0  (126.0-154.0) | **<0.001** |
| **DBP (mmHg)** | 73.0  (67.0-81.0) | 80.0  (72.0-88.0) | **<0.001** | 76.0  (69.0-84.0) | 79.0  (72.0-87.0) | **<0.001** | 79.0  (71.0-88.0) | 84.0  (76.0-92.0) | **<0.001** | 79.0  (70.0-87.0) | 82.0  (74.0-91.0) | **<0.001** |
| **BMI(kg/m^2^)** | 21.9  (19.9-24.2) | 25.9  (23.9-28.3) | **<0.001** | 22.0  (20.0-24.0) | 25.2  (23.4-27.3) | **<0.001** | 23.0  (20.6-25.4) | 26.5  (24.5-28.8) | **<0.001** | 22.9  (20.8-25.0) | 25.6  (23.9-27.6) | **<0.001** |
| **AST (IU/L)** | 17.5  (15.0-21.0) | 19.0  (16.0-24.0) | **<0.001** | 21.0  (18.0-26.0) | 20.0  (18.0-25.0) | **<0.001** | 22.0  (18.0-31.0) | 23.0  (19.0-30.0) | **<0.001** | 22.0  (18.0-29.0) | 22.0  (18.0-26.0) | **<0.001** |
| **ALT (IU/L)** | 12.0  (9.0-17.0) | 16.0  (12.0-25.0) | **<0.001** | 14.0  (11.0-20.0) | 17.0  (13.0-24.0) | **<0.001** | 22.0  (14.0-36.0) | 28.1  (20.0-43.0) | **<0.001** | 18.0  (13.0-28.0) | 21.0  (15.0-29.0) | **<0.001** |
| **GGT (IU/L)** | 14.0  (11.0-20.0) | 22.0  (16.0-32.0) | **<0.001** | 19.0  (14.0-28.0) | 24.0  (18.0-35.0) | **<0.001** | 32.0  (21.0-61.0) | 43.0  (29.0-70.0) | **<0.001** | 31.0  (21.0-54.0) | 34.0  (24.0-51.0) | **<0.001** |
| **Triglycerides (mmol/L)** | 1.0(0.7-1.5) | 1.6(1.1-2.3) | **<0.001** | 1.1(0.8-1.6) | 1.6(1.2-2.2) | **<0.001** | 1.2(0.9-1.8) | 1.8(1.3-2.7) | **<0.001** | 1.1(0.9-1.6) | 1.6(1.2-2.3) | **<0.001** |
| **HDL-C (mmol/L)** | 1.4(1.2-1.6) | 1.2(1.0-1.3) | **<0.001** | 1.3(1.1-1.5) | 1.2(1.0-1.4) | **<0.001** | 1.1(0.9-1.3) | 1.0(0.9-1.2) | **<0.001** | 1.1(0.9-1.3) | 1.0(0.9-1.2) | **<0.001** |
| **glucose (mmol/L)** | 4.4(4.1-4.8) | 4.7(4.3-5.2) | **<0.001** | 4.7(4.3-5.1) | 5.1(4.6-5.9) | **<0.001** | 4.5(4.1-5.0) | 4.8(4.4-5.5) | **<0.001** | 4.7(4.3-5.4) | 5.1(4.6-6.1) | **<0.001** |
| **Albumin (g/L)** | 39.4  (36.7-42.0) | 40.4  (38.1-42.6) | **<0.001** | 38.7  (36.0-41.2) | 39.8  (37.6-42.1) | **<0.001** | 40.8  (37.4-43.6) | 42.4  (40.1-44.6) | **<0.001** | 38.1  (34.9-40.8) | 39.8  (37.5-42.2) | **<0.001** |
| **Platelet count (10^9^/L)** | 259.0(216.0-308.0) | 293.0(247.0-343.0) | **<0.001** | 246.0(203.0-296.0) | 257.0(218.0-301.0) | **<0.001** | 242.0(198.0-291.0) | 257.0(218.0-303.0) | **<0.001** | 232.0(187.0-285.0) | 233.0(195.0-275.0) | 0.301 |
| **Elevated glucose (%)** | 1823(6.8) | 911(17.2) | **<0.001** | 3372(14.2) | 4890(31.1) | **<0.001** | 2098(13.4) | 1404(22.5) | **<0.001** | 6531(21.0) | 4159(34.2) | **<0.001** |
| **Elevated BMI (%)** | 9589(35.7) | 4514(85.2) | **<0.001** | 8737(36.7) | 12382(78.8) | **<0.001** | 7878(50.5) | 5628(90.1) | **<0.001** | 15480(49.8) | 10420(85.6) | **<0.001** |
| **Elevated triglycerides (%)** | 5427(20.2) | 2226(42.0) | **<0.001** | 4641(19.5) | 6934(44.1) | **<0.001** | 4398(28.2) | 3491(55.9) | **<0.001** | 6774(21.8) | 5513(45.3) | **<0.001** |
| **Low HDL-C (%)** | 11549(43.0) | 3807(71.8) | **<0.001** | 12044(50.6) | 10550(67.1) | **<0.001** | 5904(37.8) | 2920(46.8) | **<0.001** | 12173(39.1) | 5667(46.6) | **<0.001** |
| **Elevated BP (%)** | 6535(24.3) | 2547(48.1) | **<0.001** | 13652(57.3) | 11089(70.5) | **<0.001** | 7474(47.9) | 3952(63.3) | **<0.001** | 19837(63.8) | 9039(74.3) | **<0.001** |
| **Cigarette smoke (%)** |  |  | 0.307 |  |  | 0.806 |  |  | **<0.001** |  |  | **<0.001** |
| **Current** | 46(0.2) | 12(0.2) |  | 46(0.2) | 32(0.2) |  | 3024(19.4) | 683(10.9) |  | 6047(19.4) | 1483(12.2) |  |
| **Former** | 25(0.1) | 2(0.0) |  | 31(0.1) | 17(0.1) |  | 1180(7.6) | 231(3.7) |  | 4725(15.2) | 1038(8.5) |  |
| **Never** | 26787(99.7) | 5285(99.7) |  | 23738(99.7) | 15669(99.7) |  | 11405(73.1) | 5331(85.4) |  | 20331(65.4) | 9648(79.3) |  |
| **T2DM (%)** | 475(1.8) | 437(8.2) | **<0.001** | 2804(11.8) | 3701(23.5) | **<0.001** | 1173(7.5) | 755(12.1) | **<0.001** | 5518(17.7) | 3253(26.7) | **<0.001** |
| **CKD (%)** | 833(3.1) | 76(1.4) | **<0.001** | 1235(5.2) | 441(2.8) | **<0.001** | 1472(9.4) | 274(4.4) | **<0.001** | 3042(9.8) | 866(7.1) | **<0.001** |
| **CVD (%)** | 155(0.6) | 109(2.1) | **<0.001** | 2868(12.0) | 2708(17.2) | **<0.001** | 1026(6.6) | 593(9.5) | **<0.001** | 6720(21.6) | 3206(26.3) | **<0.001** |
| **Current drinking alcohol**  **(%)** | 125(0.5) | 0(0.0) | **<0.001** | 165(0.7) | 0(0.0) | **<0.001** | 4388(28.1) | 0(0.0) | **<0.001** | 9480(30.5) | 0(0.0) | **<0.001** |
| **FIB4(%)** |  |  | **<0.001** |  |  | **<0.001** |  |  | **<0.001** |  |  | **<0.001** |
| **<1.3** | 21653(89.9) | 4349(93.5) |  | 8449(41.6) | 7270(55.6) |  | 10969(81.8) | 4682(91.4) |  | 10810(40.1) | 5026(50.3) |  |
| **1.3-2.67** | 1897(7.9) | 234(5.0) |  | 9699(47.8) | 5210(39.8) |  | 1606(12.0) | 365(7.1) |  | 12370(45.9) | 4328(43.3) |  |
| **＞2.67** | 549(2.3) | 67(1.4) |  | 2151(10.6) | 607(4.6) |  | 831(6.2) | 76(1.5) |  | 3778(14.0) | 637(6.4) |  |

**Note: Values in bold are significant (*P* < 0.05).**

Baseline characteristics are expressed as frequencies (proportions) for categorical variables and as medians (interquartile ranges [IQRs]) for continuous variables.

The Mann-Whitney U test and the chi-square test were used to evaluate the differences in the distribution of continuous variables and categorical variables between groups, respectively. Missing data for ALT, AST, GGT (1.3% each), PLT (13%), and albumin (1.3%) due to incomplete medical records; treated as NA in the analysis.

**Abbreviations:** MASLD, Metabolic dysfunction-associated steatotic liver disease; SBP, Systolic blood pressure; DBP, Diastolic blood pressure; BMI, Body mass index; AST, Aspartate aminotransferase; ALT, Alanine aminotransferase; GGT, Gamma-glutamyl transferase; HDL-C, High-density lipoprotein cholesterol; Elevated BP, Elevated blood pressure; T2DM, Type 2 diabetes; CKD, Chronic kidney disease; CVD, Cardiovascular disease; FIB-4, Fibrosis-4 index.

**Supplementary Table S8. Multivariable logistic regression for MASLD stratified by age group**

| **Age group** | **Female vs male, adjusted OR (95% CI)** | **P value** |
| --- | --- | --- |
| 18–50 years | 0.974 (0.925–1.025) | 0.311 |
| 51–80 years | 2.342 (2.262–2.426) | <0.001 |

Note: OR, odds ratio; CI, confidence interval. Models were adjusted for age, BMI, glucose, triglycerides, HDL-C, T2DM, CVD, and calendar year. An OR > 1 indicates higher odds in females; an OR < 1 indicates higher odds in males.
